# NK cells mediate the cumulative analgesic effect of electroacupuncture in a rat model of neuropathic pain

**DOI:** 10.1186/1472-6882-14-316

**Published:** 2014-08-26

**Authors:** Yong-Hui Gao, Jun-Ying Wang, Li-Na Qiao, Shu-Ping Chen, Lian-Hong Tan, Qiu-Ling Xu, Jun-Ling Liu

**Affiliations:** Department of Physiology, Institute of Acupuncture and Moxibustion, China Academy of Chinese Medical Sciences, Beijing, 100700 China; Hainan Medical University, Haikou, Hainan Province 571101 China

**Keywords:** Acupuncture analgesia, Cumulative effect, Chronic constriction injury, Innate immunity, Acquired immunity, NK cells

## Abstract

**Background:**

Cumulating evidence has revealed the effectiveness of acupuncture therapy in relieving pain via immunoregulation. However, its underlying mechanism remains unknown. The present study was designed to determine the changes of immunogenic responses at different time-points of electroacupuncture (EA) interventions in neuropathic pain rats.

**Methods:**

The neuropathic pain model was established by ligature of the left sciatic nerve to induce chronic constriction injury (CCI). EA was applied at Zusanli (ST36) and Yanglingquan (GB34) for the EA groups. The thermal pain threshold was detected with an algesia-detector. The subgroups of plasma and splenic lymphocytes were determined via fluorescence-activated cell sorting. Specific inflammatory cytokines were assayed using an ELISA-based bead multiplex assay. The activities of splenic natural killer (NK) cells and cytotoxic T lymphocytes were detected by methyl thiazolyl tetrazolium colorimetric method. For confirming the involvement of NK cell in EA-analgesia, anti-asialo-ganglio-N-tetraosylceramide (anti-asialo-GM1) antibody was given to CCI rats before EA.

**Results:**

Following CCI, the thermal pain threshold of the affected hind footpad was significantly decreased, and increased from the 3^rd^ day to the 12^th^ day after EA interventions, presenting a time-dependent tendency from the 5th day on. From day 3 to 5 of EA interventions, the percentages and activity of splenic NK cells, concentrations of splenic interleukin-2 (IL-2) and beta-endorphin (β-EP) were significantly increased. Meanwhile, the concentrations of plasma IL-2, IL-1β and gamma-interferon (IFN-γ) were significantly decreased and returned to the normal level on day 12 following EA. Plasma transforming growth factor-β (TGF-β) levels were considerably upregulated on day 5 and 12 following EA. The CD4+/CD8+ T cell ratio was markedly downregulated compared with the control and CCI groups on day 5 and returned to the normal level on day 12 following EA. After depleting NK cells by anti-asialo-GM1 antibody, the increased thermal pain threshold following EA intervention was obviously reduced.

**Conclusions:**

Repeated EA interventions have a time-dependent cumulative analgesic effect in neuropathic pain rats, which is closely associated with its regulatory effects on NK cells, splenic IL-2, β-EP, and plasma IL-2, IL-1β, IFN-γ and TGF-β levels.

## Background

Recent data have demonstrated a critical involvement of innate and adaptive immune responses following chronic pain [1, 2]. A pivotal event that defines the successful outcome of any inflammatory or injury event is the transition from innate to acquired immunity [3]. The innate immune response is the first line of defense against alarm signals from the injured tissue. The modulation of the innate immunological response to chronic pain has emerged as a promising therapeutic target [4]. T-lymphocyte recruitment plays a role in the establishment of pain chronicity (between days 3–21). In some patient cohorts, antibodies to neuronal antigens have been reported [5, 6]. Therefore, acquired immunity may also be a good biomarker for the management of clinical neuropathic pain [4, 7].

The clinical practice of acupuncture therapy has received worldwide attention as a strategy to attenuate the symptoms of pain [8–10]. Experimental studies have shown that acupuncture intervention effectively relieves chronic neuropathic pain and inflammatory pain in rats [11, 12]. In the last decade, compelling evidence has demonstrated that the anti-nociceptive effect of electroacupuncture (EA) is closely associated with immunomodulation [13, 14]. Cumulative evidence revealed that EA intervention could enhances immune function after surgical trauma or neuropathic pain in human [15] and in animals [16, 17]. However, the mechanism underlying immunoregulation of EA intervention is not clear.

To relieve pain and maintain the efficiency of EA intervention in clinical practice, successive courses of EA treatment are usually necessary for patients with chronic pain. In our previous studies, repeated EA intervention induces a cumulative effect in chronic constriction injury (CCI) rats [18–20]. The analgesic effect of EA peaked after several successive sessions of treatment. This characteristic may be similar to activation of the acquired immune reaction from innate immune response. Therefore, we hypothesized that the cumulative effect of repeated acupuncture intervention may be related to the transition of the innate immune response to the acquired immune response. Thus far, several studies have already examined the effect of EA on innate immune factors. Kim and colleagues [17] demonstrated that the activity of splenic natural killer (NK) cells which are important cellular components of the innate immune response is increased after EA via the induction of cluster of differentiation 94 (CD94). EA also effectively reverses the deleterious immunological changes in the postoperative state by regulating the balance of help T (Th)1/Th2 cytokines, thereby influencing the acquired immunity [21, 22]. However, only few studies have explored the dose–response relationship between the acupuncture stimulation and the change of immunomodulation.

In the present study, we set out to examine the change of acupuncture-associated immunoregulation under neuropathic pain conditions. Specifically, we assessed the role of different lymphocyte subgroups, cytokines, and mediators of analgesia at different time-points of EA interventions to gain insights into its mechanism underlying the conversion from innate to acquired immunity.

## Methods

### Animals and grouping

A total of 50 male Wistar rats (240–300 g) were obtained from the Experimental Animal Center of Peking Union Medical College (Beijing, China), and housed within the animal care facilities in the Institute of Acupuncture and Moxibustion, China Academy of Chinese Medical Sciences. Animals were randomly assigned (n = 10 in each group) to sham ligature (CON), CCI pain model (CCI) and CCI plus three, five, and twelve sessions of EA treatment (EA3d, EA5d, and EA12d, respectively) groups. In order to observe the involvement of NK cells in EA analgesic effect, EA + Anti-asialo-ganglio-N-tetraosylceramide (anti-asialo-GM1) (EA + anti-asialo-GM1) group and EA + normal rabbit serum (EA + serum) group were added. All experimental procedures were approved by the Institute of Acupuncture and Moxibustion of China Academy of Chinese Medical Sciences, and performed according to the “*Guidelines for Laboratory Animal Care and Use*” of the Chinese Ministry of Science and Technology (2006).

### CCI pain model and pain threshold detection

The CCI model was established by ligating the unilateral sciatic nerve, as previously reported [23]. Briefly, under anesthesia (25% urethane plus 1.5% chloralose, 0.4 mL/100 g body weight) and routine sterilization, the left sciatic nerve was exposed at the mid-thigh level by blunt dissection through the biceps femoris. Four constrictive ligatures (4–0 non-absorbable suture) were tied around the nerve at the distal end close to the bifurcation site (about 1 mm space between every two ligatures). The ligature was all right until a moderate muscular contraction of the leg was seen. The same procedure was performed for rats in the CON group but without nerve ligature.

The paw withdrawal latency (PWL) (i.e. the thermal pain threshold) of the bilateral hind paws was determined using a 37370 Algesia Detector (Ugo, Italy) 5 days after CCI, and every day thereafter prior to EA treatment. The radiant heat source was focused on the plantar surface of the hindpaw, and light intensity was preset to obtain a baseline latency of approximately 15 sec. Each rat underwent two trials with a five-min interval, and the mean value of the two trials was used as the PWL. To minimize differences in individual animals, the difference value of PWL (PWLD) between the healthy and the affected hindpaws was calculated.

### EA intervention

According to the theory of traditional Chinese medicine, Zusanli (ST36) and Yanglingquan (GB34) are considered to be the most effective acupoints for treating lower back pain, and are commonly used in modern research to study the effects of acupuncture on various physiological regulatory and control systems. ST36 is located 5 mm beneath the capitulum fibulae and lateral-posterior to the knee-joint. GB34 is approximately 5 mm superior-lateral to ST36. In the present study, 7 days after CCI, animals from the three EA groups were stimulated with EA at bilateral ST36 and GB34. The acupoints were punctured with stainless steel filiform needles (diameter 0.35 mm, length 40 mm, Huatuo; Suzhou Medical Appliance Manufactory, Jiangsu, China) to a depth of approximately 2–3 mm, and stimulated electrically for 30 min using a Han’s EA Stimulator (LH202; Neuroscience Research Center, Peking University, Beijing, China). The intensity and frequency of EA were 1 mA and 15 Hz, respectively. During EA stimulation, the animals were conscious and constrained with a special cloth bag. The treatment was administered once daily continuously for 3, 5 or 12 consecutive days. The other two groups underwent the same procedure but without EA stimulation.

At the end of the EA treatment, all rats from the five groups were deeply anesthetized. Blood samples (3–5 mL) were obtained through carotid intubation and placed separately in three heparinized tubes. Spleen and hypothalamus tissues were rapidly removed on an ice plate and weighed.

### Lymphocyte preparation and fluorescence-activated cell sorting (FACS)

Spleen tissue was washed with 5 mL RPMI-1640 medium (Gibco, Grand Island, USA) and then minced with two sterilized glass slides, passed through a 70-μm nylon cell strainer, and washed. The suspension was differentially centrifuged over a Percoll gradient (EMB Biosciences, La Jolla, CA) (40% Percoll, 1200 r/min, 10 min, 25°C). The lymphocytes were washed with phosphate-buffered saline (PBS), and then suspended in RPMI-1640 medium, and 10^6^ single cell suspensions were prepared.

Whole blood (100 μL) and splenic lymphocytes (10^6^) were triple-stained with (1) APC-anti-CD3, FITC-anti-CD45RA and PE-anti-CD161a, or (2) APC-anti-CD3, PE-anti-CD4 and FITC-anti-CD8a (all from BD Biosciences, San Jose, CA, USA) for 30 min at 4°C in the dark, then washed with PBS and resuspended in FACS staining buffer (BD Biosciences). Three-color analysis was performed on a FACSCalibur (BD Biosciences) with a 100,000–200,000 event count. Data were analyzed using FlowJo software (TreeStar, San Carlos, CA, USA).

### Multiplex enzyme-linked immunosorbent assay (ELISA) analysis of plasma and splenic cytokine concentrations

To prepare splenic aqueous extract for the cytokine concentration assay, the spleen was removed rapidly on an ice plate, and homogenized by a glass tissue homogenizer in an ice cold water bath. The homogenate was then centrifuged (4500 r/min for 30 min, at 4°C). The supernatant was collected and stored at -80°C until future use. Plasma was separated by centrifugation (3000 r/min for 30 min, at 4°C) and stored at -80°C until use. Concentrations of plasma gamma-interferon (IFN-γ), interleukin-1 beta (IL-1β), IL-2, IL-6, IL-4, IL-10, and transforming growth factor beta (TGF-β), and splenic IL-2, IL-12, IL-15 and IFN-γ were simultaneously quantified in samples using an ELISA-based bead multiplex assay (Linco Research, St Charles, MO, USA), according to the manufacturer’s instruction. Samples were analyzed with a Lumine × 100 plate reader (Linco Research, St Charles, Missouri, USA) to determine the cytokine concentrations. Concentration of each cytokine in the multiplex assay was calculated from calibration curves using individual recombinant proteins as standards. All specimens were tested in duplicate wells to assess inter-assay variability.

### NK cell and cytotoxic T lymphocyte (CTL) activities assay

Splenic CD8^+^ T cell subset and NK cells were isolated using MagCellect Rat CD8^+^ T Cell Isolation Kit (R&D Systems, Minneapolis, MN) and Rat NK Cell Isolation Kit (Yanjin Biotec, Shanghai, China) according to the manufacturer’s instructions. The activities of NK cells and CTLs were respectively detected by methyl thiazolyl tetrazolium colorimetric (MTT) method [24]. Briefly, NK cells of each group were collected and seeded in 96 well plates as effect cells at a concentration of 1 × 10^5^/ml and cultured 5 × 10^5^ YAC-1 used as target cells. Co-cultured NK and YAC-1 cells in 96-well plate at a 50:1 ratio of effector to target (E:T) were simultaneously incubated at 37°C in 5% CO_2_ for 4 hours in 96 well plates. Then the supernatants were discarded and fresh medium containing 200 μL MTT was added to each well and incubated for 4 hours. After the supernatants were discarded, 20 μL dimethylsulfoxide was added to each well and mixed thoroughly to dissolve the blue crystals at room temperature. Ten minutes later, when all the crystals dissolved, the plates were read on a plate reader (Bio-Rad, Hercules, CA, USA) at 560 nm. For CTL activity, the effector cells were 2.5 × 10^6^/ml splenic CD8^+^ T lymphocytes and cultured SP2/0 (5 × 10^5^) cells used as target cells. Co-cultured CD8+ T and SP2/0 cells at a 50:1 ratio of effector to target (E:T) were incubated for 24 hours. All MTT assays were performed in triplicate. The activities of CTL and NK cell were calculated using the following formula: NK (or CTL) activity = [1-(OD (E + T)-ODE)/ODT] × 100% (ODE, optical density of effect cells; ODT, optical density of target cells; OD (E + T), optical density of co-cultured cells).

### Beta-endorphin (β-EP) assay

The hypothalamus tissue samples were placed in tubes containing 1 mol/L (0.5 mL) glacial acetic acid to be homogenized on ice. The tissue lysate was centrifuged (3500 r/min, at 4°C, for 20 min). The concentration of β-EP was measured by the β-EP ^125^I radioimmunoassay kit for rat (Sino-UK Institute of Biological Technology, Beijing, China) according to the instruction. All specimens were tested three times to minimize inter-assay variability.

### Depletion of NK cells in vivo

For observing the effect of NK cells on EA analgesia in CCI rats, other 20 Wistar rats were equally divided into EA + normal rabbit serum group and EA + Anti-asialo-GM1 rabbit antibody group. The procedures of CCI and EA were the same to those mentioned above. The Anti-asialo-GM1 rabbit antibody (Abcam Cambridge, UK) was intravenously given at a dose of 80 μg on the day before the first EA treatment and once again every 4 days thereafter. Normal rabbit serum was used as control.

### Statistical analysis

All data are presented as the mean ± standard deviation (SD), and were analyzed by one-way analysis of variance followed by Fisher-Hayter for multiple comparisons with Stata 12.0 software (Stata Corp, College Station, USA). All *P* values were 2-sided, and *P* < 0.05 was considered significant.

## Results

### Effects of successive EA on pain threshold

**Figure 1:**
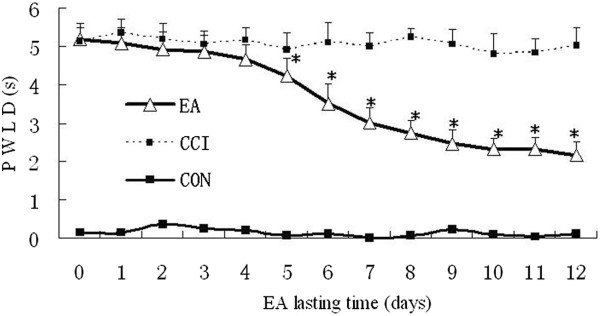
**Effect of EA stimulation on PWLD in rats with neuropathic pain (n = 10 in each group).** PWLD was recorded before and during days 1–12 of EA intervention. Data are presented as the mean ± SD. * *P* < 0.05 vs the CCI group.

No significant differences were found between CON, CCI model and CCI + EA groups in the PWLD before CCI (data not shown). After CCI surgery, rats showed typical neuropathic behavioral responses and a significantly shortened PWL on the nerve ligature side (*P* < 0.05). PWLD was considerably increased in the CCI group compared with the CON group (*P* < 0.05). After three daily sessions of EA, the PWLD was gradually decreased. From the 5^th^ session on, PWLD was significantly lower than that of the CCI group (*P* < 0.05) (Figure 1), thereby presenting a time-dependent effect. It suggests an apparent cumulative analgesic effect of repeated EA intervention.

### Effect of different sessions of EA on lymphocytic subpopulations

**Figure 2:**
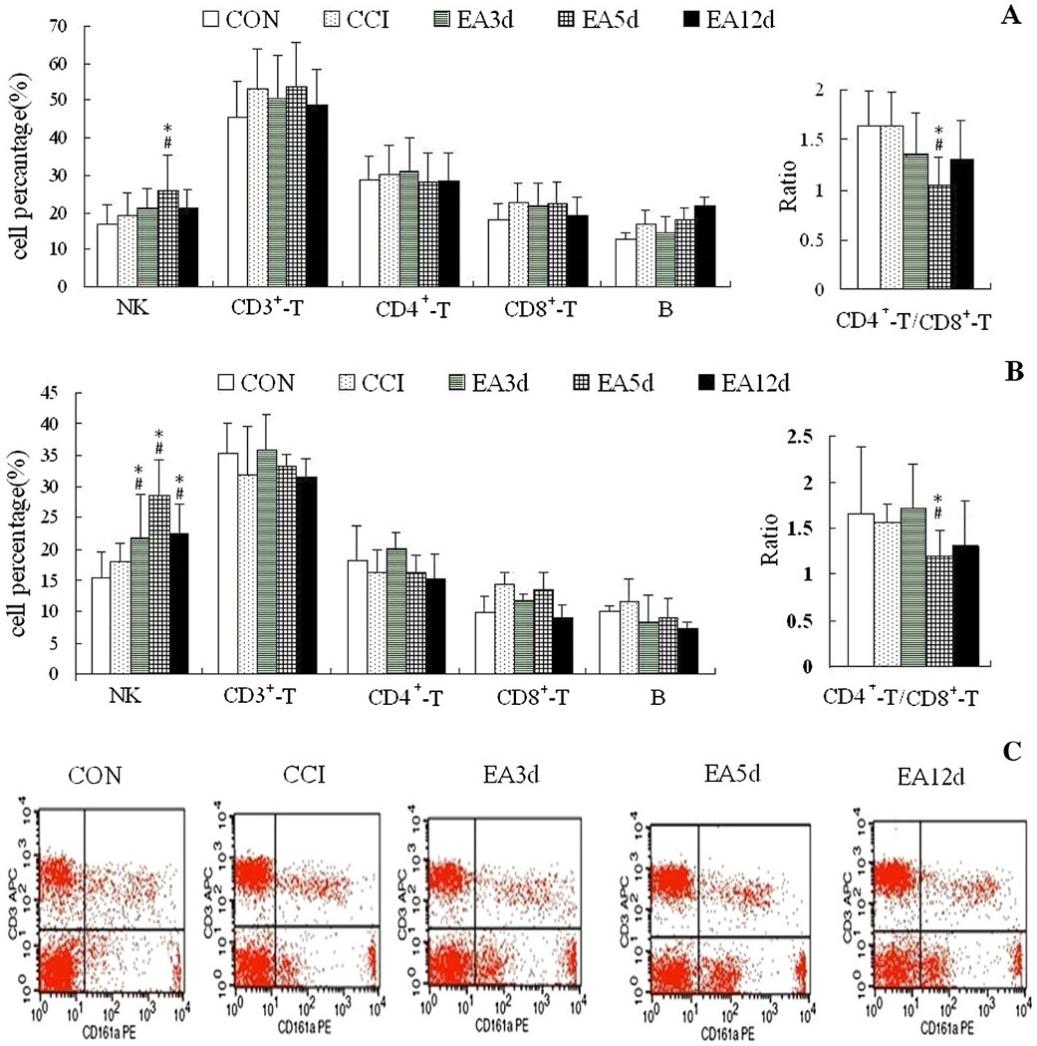
**Effect of EA on levels of splenic lymphocytic subgroups (A) and plasma lymphocytic subgroups (B) and a representative FACS scan image of NK cells (C).** Data are presented as the mean ± SD (n = 10 per group). ^#^ *P* < 0.05 vs the CON group, *P < 0.05 vs the CCI group.

Splenic NK cell percentages of the EA5d group, plasma NK cell percentages of the EA3d, EA5d and EA12 d groups were significantly increased compared with the model group (*P* < 0.05). The CD4^+^/CD8^+^ ratio of the EA5d group was markedly (*P* < 0.05) downregulated compared with the CON and CCI groups (Figure 2).

### Effect of repeated EA intervention on splenic NK and CTL cell activities

**Figure 3:**
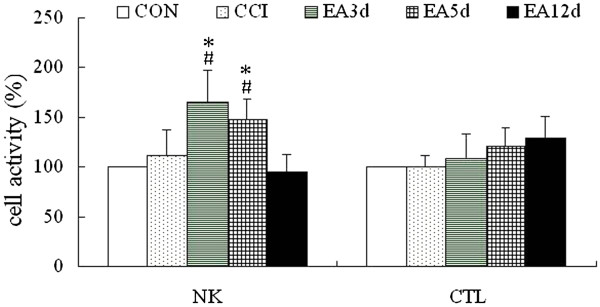
**Effect of repeated EA on splenic NK cell and CTL cell activities.** Data are presented as the mean ± SD (n = 10 per group). ^#^ *P* < 0.05 vs the CON group, *P < 0.05 vs the CCI group.

The above-mentioned results showed that the percentage of NK cell was increased and the CD4^+^/CD8^+^ ratio decreased following EA intervention. CTL is mainly CD8+ T cell and an effector cell in cell immunity. Therefore, we investigated NK and CTL activities to better understand the underlying mechanism of these changes. The results showed that NK cell activity was significantly increased in the EA3d and EA5d groups compared with the CCI group (*P* < 0.05) and returned to the control following 12 sessions of EA. No obvious change in CTL activity was detected in the five groups (Figure 3).

### Effect of EA on NK cell activation-associated factors

**Figure 4:**
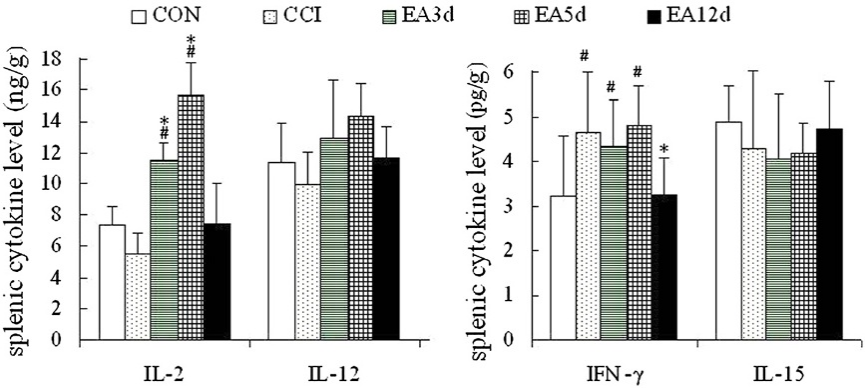
**Effect of EA on NK cell activation-associated cytokines.** Data are presented as the mean ± SD (n = 10 per group). ^#^ *P* < 0.05 vs the CON group, *P < 0.05 vs the CCI group.

To elucidate the mechanism underlying the increase in NK cell activation, NK cell activation-related cytokines were assayed. Splenic IL-2, IL-12 and IL-15 concentrations were slightly but not significantly down-regulated in the CCI group compared with the CON group. The concentration of IFN-γ was significantly increased in the CCI and the EA5d groups (*P* < 0.05). After EA intervention, IL-2 levels of the EA3d and EA5d groups were markedly increased compared with the CCI group (*P* < 0.05). The concentration of splenic IFN-γ in the EA12d group was notably reduced to normal level (*P* < 0.05, compared with the CCI group) (Figure 4).

**Figure 5:**
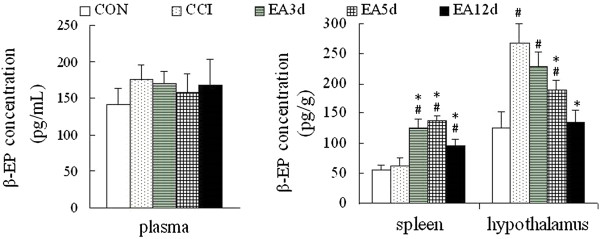
**Effect of EA on β-EP concentration.** Data are presented as the mean ± SD (n = 10 per group). ^#^ *P* < 0.05 vs the CON group, *P < 0.05 vs the CCI group.

β-EP has also been reported to increase NK cell activity [25]. Therefore, we investigated its concentration in plasma, spleen and hypothalamus. Hypothalamic concentration of β-EP was dramatically increased after CCI (*P* < 0.05), which did not occur in plasma and spleen. Hypothalamic β-EP concentration was notably reduced in the EA3d and EA5d groups (*P* < 0.05), while splenic β-EP levels in EA3d, EA5d, and EA12d groups were considerably upregulated (*P* < 0.05, Figure 5).

### Effect of EA on plasma pro-inflammatory and anti-inflammatory cytokines

**Figure 6:**
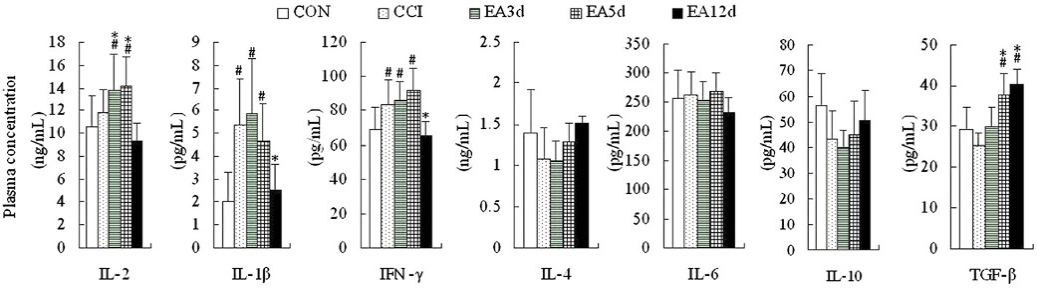
**Effect of EA on plasma cytokine concentrations in CCI rats.** Data are presented as the mean ± SD (n = 10 per group). ^#^ *P* < 0.05 vs the CON group, *P < 0.05 vs the CCI group.

To further elucidate changes of the immune response at different acupuncture intervention time-points, pro-inflammatory cytokines and anti-inflammatory cytokines in the plasma were examined. In comparison with the control group, plasma pro-inflammatory cytokine concentrations were apparently (IL-1β, IFN-γ, *P* < 0.05) or mildly (IL-2, *P* > 0.05) increased in the CCI group. Anti-inflammatory cytokines, IL-4, IL-10 and TGF-β were slightly but not significantly downregulated (Figure 6).

Plasma IL-1β and IFN-γ levels of the EA 12d group were markedly downregulated to normal level (*P* < 0.05). In the same group, IL-4 and IL-10 levels were moderately yet not significantly upregulated. TGF-β levels in the EA5d and EA12d groups were considerably upregulated (*P* < 0.05), suggesting that repeated EA treatment inhibit inflammation.

### Effect of anti-asialo GM1 on pain threshold following EA

**Figure 7:**
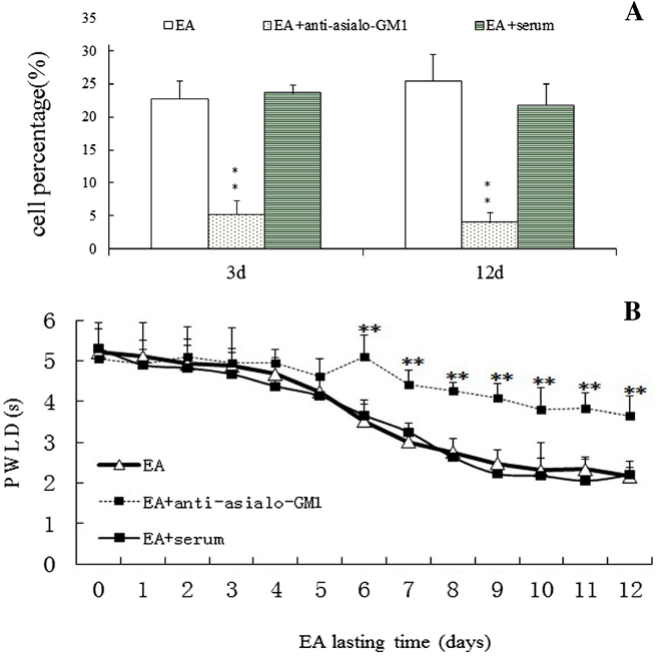
**Effect of anti-asialo GM1 antibody on plasma NK cell population (A) and on EA stimulation induced reduction of PWLD in rats with neuropathic pain (B).** ** *P* < 0.05 vs the EA and EA + serum groups.

To confirm the effect of NK cells on EA analgesic, we used anti-asialo GM1 to abolish NK cells in vivo before EA treatment to observe the change of PWLD. As shown in Figure 7, after depleting the NK cell, the decrease of PWLD was not as obvious as the EA group and showed significant difference with EA group from the 6^th^ day on following EA intervention. These results suggest that splenic and plasma NK cells participated in the repeated EA intervention-induced cumulative analgesic effect in CCI rats.

## Discussion

Although no single theory has successfully offered a unified physiological explanation for the effects of acupuncture, some researchers have proposed that its analgesic action may involve regulation of the body immunity [26]. In the present study, the PWLD was not changed at the early stage of EA (days 1–3), but increased considerably after 4–5 sessions of EA intervention and presented a time-dependent manner. This result demonstrated that the body’s response to EA at the early stage may be different from that at the “cumulative” stage. Under CCI, the “cumulative” effect commenced from 4–5 days following EA. Interestingly, this point is also the shifting time from innate immunity to acquired immunity. Therefore, we selected the time points of day 3 and 5 following EA to examine the change of immune regulation from lymphocyte subgroups and related cytokines.

We observed that lymphocyte subpopulation NK cell was increased both in their activity and in quantity in the spleen and peripheral blood following EA intervention. Next, we examined changes of the NK cell activation-associated factors following acupuncture treatment to elucidate the mechanism of the increase in NK cells. IL-2 and β-EP, two efficacious activators of NK cells, were increased. Some studies have shown that the effect of β-EP on NK cell activity is dependent on its location [27]. Our results demonstrated that in the hypothalamus, the β-EP mainly functions as an analgesic substance. However, in the spleen, its role is complex because of a possible combination with IL-2 to enhance NK cell activity. These results confirmed that NK cell may be an effector at the early stage of EA to initiate its cumulative effect, which was evidenced by the studies of Yu [28, 29].

It has been demonstrated that loss of larger myelinated fibers in a CCI model causes changes in the neurogenic component, which induced a recruitment of immune cells at the injury site of the peripheral nerve. Thus, changes in the profile of neurochemicals involving neuropathic pain affect the number and activity of immune cells in the plasma or spleen [30]. Trauma, surgical stress and chronic pain are often associated with immune suppression and increased susceptibility to infection [21]. Sunagawa’ study showed that splenic NK cell activity was suppressed by ligation of unilateral mental nerve in rats [31]. NK cells are the third subset of lymphocytes and are important for the early immune response against infections. It thus appears to be important to prevent the relative suppression of NK cell to maintain immune function. Our study suggests that EA stimulation has a positive role in the immunoregulation after CCI through increasing the NK cell function. We also observed that the ratio of CD4+/CD8+ T cell was decreased following five sessions of EA. This may suggest a change in the levels of some T-lymphocyte subgroups. However, we did not find the exact source of this change because of the wide varieties of the subgroups. T lymphocyte may have a role in the maintenance of neuropathic pain because T-lymphocyte deficient mice were found to have a reduction in injury-induced hypersentivity but adoptive transfer reversed this effect [32]. At the same time, T lymphocyte that infiltrated into the central nervous system interacted with microglia and helped maintain neuropathic pain by the secretion of pro-inflammatory mediators and also possibly by facilitating the activation of astrocytes [33]. Moreover, some studies also suggest that modulation of T- lymphocyte phenotype might be one approach to the treatment of neuropathic pain [1]. Therefore, further experiments to explore the exact changes in T cell subgroups (Th cell, Tc cell, Th17, Treg cells…) following CCI and EA should be performed.

Cytokines have emerged as essential regulators of lymphocyte trafficking, which is necessary to turn an innate immune response into an adaptive response [34, 35]. IFN-γ, IL-2 and TGF-β are all cytokines that support cellular immunity. After three sessions of EA intervention, and particularly following five successive sessions, the concentrations of IL-2 and TGF–β in the spleen or plasma were increased, suggesting an activation of adaptive immunity. Interestingly, NK cells can furthermore function as “adaptive effectors” once activated by T cell-induced IFN-γ or when IgG eventually elicits antibody-dependent cell cytotoxicity [36–38]. Therefore, NK cells not only synergize with innate immunity against danger signals but also shape the adaptive immune system [39], thus highlighting its importance as an important adjuvant during the development of immunoregulation after acupuncture intervention. NK cells are also an important source of IFN-γ which induces the production of chemokines (Interferon-inducible Protein 10, IL-8, etc.) downstream of toll-like receptor (TLR) activation from resident tissue cells [40]. These chemokines can attract antigen-specific CD4^+^ or CD8^+^T lymphocytes into the injured tissue to establish organ-specific immunity, which constitutes an important link between the innate and adaptive immune responses. This link is essential for protective immunity. However, the type of cellular immunity that occurred in the present study remains elusive because we did not examine the subpopulations of B cells and subpopulations of Th and CTL cells, which eventually elicit an “elimination” effect on danger signals. In the present study, CCI increased plasma concentrations of the pro-inflammatory cytokines, IFN-γ, IL-1β, and IL-2. However, no notable changes were seen in the levels of the anti-inflammatory cytokines, IL-4, IL-6 and IL-10. These results also suggested an imbalance of TH1/TH2. Following 12 sessions of EA, plasma levels of IL-1β and IFN-γ were significantly decreased, and the anti-inflammatory TGF-β was considerably increased, implying that after undergoing approximately 10 days of cellular immunity, the balance between TH1 and TH2 was restored.

The innate and adaptive immune systems are clearly two interdependent parts of a single integrated immune system. The innate immune response not only provides the first line of defense against “danger”, but also provides the biological context of the “danger signal” that determines which antigens the acquired immune system responds to and the nature of that response to instruct the adaptive immune system to mount a response. In the present study, the most notable decrease in PWLD occurred between day 3 and 5 of EA interventions. This time point was consistent with that of the innate immunity to motivate the acquired immunity. Moreover, following five sessions of EA, the ratio of CD4 + T/CD8+ T cell was decreased, which may suggest a change in the levels of some subgroups of CD4+ or a downregulation of subgroups of CD8+, or a combination of the two. These cells are acquired immunity response cells. These coincidences may suggest that the different stage of EA interventions was related to the shift from the innate to the acquired immune responses. However, more sophisticated researches are definitely necessary for supporting this hypothesis.

## Conclusions

Successive EA interventions have a time-dependent cumulative analgesic effect in neuropathic pain rats, which is closely associated with its regulatory effects on NK cells, splenic IL-2, β-EP, and plasma IL-2, IL-1β, IFN-γ and TGF-β levels. As an effect cell involving this cumulative analgesic effect, NK cell may be a functional bridge between the cumulative analgesic effect of EA and the immune activity and may be a target for EA analgesia. Results of the present study provide the first insight into the origins of innate to adaptive immunity following EA interventions in a rat model of neuropathic pain.

## Abbreviations

CCI: Chronic constrictive injury
EA: Electroacupuncture
CON: Normal control
PWL: Paw withdrawal latency
PWLD: Difference value of PWL
NK: Natural killer
β-EP: Beta-endorphin
CD: Cluster of differentiation
Th: Help T cell
FACS: Fluorescence-activated cell sorting
IFN-γ: Gamma-interferon
IL: Interleukin
TGF-β: Transforming growth factor beta
ELISA: Enzyme-linked immunosorbent assay
CTL: Cytotoxic T lymphocyte
MTT: Methyl thiazolyl tetrazolium.
